# Ventricle-Specific Biomechanical Responses to Inotropic and Vasoactive Drugs in Human Myocardial Slices

**DOI:** 10.1007/s12265-026-10773-9

**Published:** 2026-05-11

**Authors:** Sanne J. J. Langmuur, Jorik H. Amesz, Rahi S. Alipour Symakani, Kevin M. Veen, Christiaan L. Meuwese, Ad J. J. C. Bogers, Natasja M. S. de Groot, Olivier C. Manintveld, Yannick J. H. J. Taverne

**Affiliations:** 1 Translational Cardiothoracic Surgery Research Lab, Department of Cardiothoracic Surgery, Erasmus Medical Center, P.O. Box 2040, 3000CA Rotterdam, the Netherlands; 2 Transplant Institute, Erasmus Medical Center, Rotterdam, the Netherlands; 3 Department of Experimental Cardiology, Erasmus Medical Center, Rotterdam, the Netherlands; 4 Department of Cardiothoracic Surgery, Erasmus Medical Center, Rotterdam, the Netherlands; 5 Department of Intensive Care Adults, Erasmus Medical Center, Rotterdam, the Netherlands; 6 Translational Electrophysiology, Department of Cardiology, Erasmus Medical Center, Rotterdam, the Netherlands; 7 Department of Cardiology, Erasmus Medical Center, Rotterdam, the Netherlands

**Keywords:** Living myocardial slices, Right ventricle, Heart failure

## Abstract

This study aimed to compare baseline biomechanical contractile properties between right (RV) and left ventricular (LV) human living myocardial slices (LMS) and explore the effect of dobutamine, noradrenaline, adrenaline, levosimendan and enoximone in both ventricles. Sixty-three slices (33 RV, 30 LV) were produced from 5 explanted hearts. At baseline, RV LMS had a higher maximum contraction force, shorter contraction duration, time to peak and time to relaxation, steeper dF/dt and a larger peak area than LV LMS. Dobutamine administration had a larger effect in RV than LV LMS. Spontaneous, irregular contractions were observed after noradrenaline, adrenaline and dobutamine administration. In conclusion, RV LMS showed a stronger contraction with higher contraction velocity as compared to LV LMS and exhibited a greater response to dobutamine. These findings contribute to the understanding of the RV physiology as a separate entity, advocating for a different approach in treating RV or LV dysfunction.

**Graphical Abstract:**

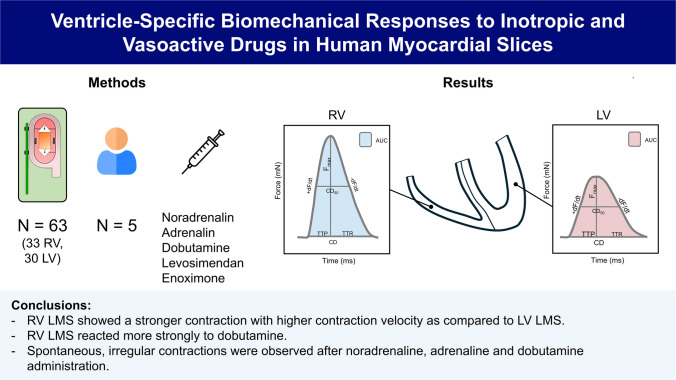

**Supplementary Information:**

The online version contains supplementary material available at 10.1007/s12265-026-10773-9.

## Introduction

Traditionally, the right ventricle (RV) has been viewed as the “low pressure bystander” to the left ventricle (LV), even though the ventricles are profoundly different [1]. Although some aspects differ between the treatment of RV and LV failure – such as management of pre- and afterload and different mechanical circulatory support strategies – current inotropic support options for RV dysfunction do not take into account the important differences between the ventricles and their use is primarily based on the effects on the LV [2].

Differences between the RV and LV start in the early stages of cardiac development. Whereas the RV is derived from the secondary heart field, the LV is developed from the primary heart field, with differences in presence of specific genes [1–5]. Furthermore, the RV has a different architecture, contraction pattern, and vascular supply compared to the LV, leading to unique biomechanical characteristics [1, 2, 6]. The RV is more suited to accommodate changes in volume loading, whereas the LV is designed to build-up pressure [1, 7]. This translates into specific anatomical features: the RV is thin-walled and has a triangular (longitudinal) and crescent (transversal) shape, as opposed to the thicker LV which has a more cylindrical shape [1]. The energy expenditure of the RV is also only about one fifth of the LV and the effects of altered loading conditions or ischemia differ between both ventricles [1].

Thus far, scarce ventricle-specific biomechanical analyses have been performed and it has been difficult to assess the direct response to medication separately in both ventricles. In patients, it is difficult to determine whether the effect of a drug is local or systemic. The same holds true for animal studies, necessitating a more fundamental research approach. In traditional in vitro models, isolated RV and LV cardiomyocytes can be cultured separately, but these models are subject to their own limitations [8]. Cellular models lack an intact extracellular matrix, 3D micro-architecture, proper cell–cell interactions and are subject to damage and dedifferentiation [9–11].

In order to allow for selective ventricle-specific functionality testing, we have proposed the use of a novel translational model using living myocardial slices (LMS) of the RV and LV [9, 11]. With this new technique, cardiac tissue from patients undergoing cardiac surgery can directly be used to create LMS from the RV and LV from the same patient. The advantage of this model is that LMS maintain 3D micro-architecture and that the observed effects can directly be related to the separate ventricles. Therefore, the objectives of this study were 1) to determine differences in biomechanical contraction profiles between RV and LV LMS, and 2) to compare the direct biomechanical response of commonly used inotropic and vasoactive drugs between the ventricles.

## Methods

### Tissue Collection

Myocardial specimens from the RV and LV lateral wall were collected from residual material during transplantation procedures. Specimens were either collected from the explanted heart of heart transplantation recipients, or from lung transplant donors whose heart was not allocated. Myocardial specimens were obtained from both RV and LV of the same patient and were immediately submerged in 4⁰C Tyrode’s solution (NaCl 136 mM, KCl 5.4 mM, MgCl2⋅6H2O 1 mM, NaH2PO4⋅H2O 0.33 mM, Glucose 10 mM, CaCl2⋅2H2O 0.9 mM, 2,3-butanedione monoxime 30 mM, HEPES 5 mM, pH 7.4). Use of this material for research purposes was approved by the Medical Ethics Committee of the Erasmus Medical Center (MEC-2020–0988).

### LMS Production and Cultivation

The preparation of LMS has been described in detail before [12–14]. In short, myocardial specimens were prepared by dissecting them to a size of about 1 cm^3^ and removing excess epicardial fat and endocardial trabeculae. Tissue blocks were then submerged in 37⁰C low-melting 4% agarose (Agarose II, VWR Chemicals LLC, Solon, OH, USA) and mounted on a precision-cutting vibratome (VT 1200S, Leica Biosystems, Nussloch, Germany). Slices of 300 μm were prepared at a blade advance speed of 0.07 mm/s and horizontal vibration amplitude of 1.30 mm, whilst submerged in 4⁰C Tyrode’s solution.

Subsequently, the tissue slices were cut to size and plastic triangles were glued to the ends in longitudinal direction, using histoacryl (B. Braun SE, Melsungen, Germany). Slices were then mounted inside a biomimetic cultivation chamber (BMCC) (InVitroSys GmbH, Munich, Germany) [12], filled with 2.4 mL 37⁰C culture medium (Gibco Medium-199 (Grand Island, NY, USA) supplemented with 5% penicillin–streptomycin, 5% insulin-transferrin-selenite and 50 µM 2-mercaptoethanol). BMCCs were placed inside a standard 37⁰C 5% CO_2_ incubator and positioned on a rocking plate for continuous medium agitation. Preload was set to 1 mN and electrical field stimulation (output 50 mA, pulse duration 7.0 ms) was immediately started at a stimulation interval of 2000 ms. One hour after start of cultivation,1.6 mL of medium was replaced, preload was readjusted to 1 mN and LMS were cultured overnight.

### Experimental Protocol

Experiments were performed the day after slice production. First of all, all medium (2.4 mL) was replaced with 2.0 mL fresh medium and preload readjusted to 1.0 mN. After one hour, baseline measurements were performed, including determination of the functional refractory period (FRP) using a pacing protocol as described below. Afterwards, the medication experiments were initiated.

**Fig. 1:**
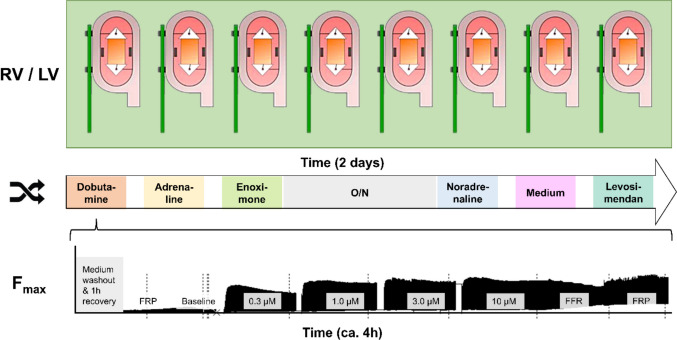
Schematic overview of the experimental protocol with eight biomimetic cultivation chambers in which six different drugs were added in a varying order over two days. For each drug, a pacing and titration protocol was followed as shown in the bottom figure. FFR force frequency relationship, FRP functional refractory period, LV left ventricle, O/N overnight, RV right ventricle

Five inotropic drugs were used in this study: noradrenaline (Centrafarm, Breda, the Netherlands), adrenaline (Apotheek A15, Gorinchem, the Netherlands), dobutamine (Centrafarm, Breda, the Netherlands), levosimendan (Simdax, Orion Pharma, Mechelen, Belgium) and enoximone (Perfan, Carinopharm GmbH, Eime, Germany). Every LMS was subjected to incremental dosages of different medications. This was done according to a prespecified protocol and in a structured order, with a different predefined starting drug for each LMS to account for the potential influence of cultivation time (Fig. 1).

Each medication was diluted in culture medium, such that by adding 4 µL, concentrations of 0.3 µM, 1 µM, 3 µM, and 10 µM were subsequently established in the BMCC. Each concentration was maintained for 20 min, then the next concentration was added. In addition to the five medications, one control run with only 4 µL of culture medium was performed. At the 10 µM dosage, the FRP and force-frequency relationship (FFR) were determined after 20 min.

After these stimulation protocols, all medium (2.0 mL) was replaced and preload readjusted to 1 mN. LMS were left to recover for one hour before starting the following experiment round with a subsequent drug. This way, three rounds of medication experiments were performed on the first day after slice production, and three on the second day. At the start of the second day, all medium was replaced again and preload adjusted, one hour before start of experimentation, equivalent to the first day.

### Functional Refractory Period

FRP was assessed by providing stimulation with a fixed pacing stimulus (S1) every 2000 ms and applying additional pacing stimuli (S2) with a decreasing interval to the S1 stimulus, from 960 to 140 ms with decremental steps of 20 ms. The first S2 stimulus that did not result in contractile capture was determined as the FRP.

### Force-Frequency Relationship

The FFR was investigated by decreasing the fixed pacing interval in the following order: 2000 ms—1000 ms—667 ms—500 ms—400 ms—333 ms. Each interval was maintained for one minute. An FFR plot was created by extracting the last 30 s of data at one frequency and plotting the average maximum force amplitude against that specific frequency.

### Irregular Contractions

Irregular contractions were registered if the LMS had spontaneous contractions independent of the electrical stimulus for > 10 s at 10 µM. All approx. 1 h recordings at the 10 µM dose were assessed for irregular contractions that could be clearly distinguished from baseline noise by one of two observers (SL and JA), with any uncertain cases discussed and resolved by consensus.

### Data Processing

Data was recorded with MyoDish Software [12] and processed with the peak analysis module of Labchart 8 (AD Instruments). The maximum force amplitude (F_max_), peak area (AUC), contraction duration (CD), contraction duration at 50% of the maximum amplitude (CD_50_), time to peak (TTP), time to relaxation (TTR), steepest positive slope (+ dF/dt), and steepest negative slope (− dF/dt) were extracted. For each LMS, means were calculated over a period of 30 s, at baseline and at the maximum increase in F_max_ after administering each different dose. The half maximal effective concentration for each drug (EC_50_) was determined using dose–response curves that were constructed using F_max_.

LMS were excluded from analysis if the culture medium was contaminated during experiments, irregular and spontaneous contractions occurred at the data extraction timepoint, or when it was impossible to distinguish the contractions from baseline rocker noise.

### Statistical Analysis

Statistical analysis and creation of all figures was performed using Microsoft Office Excel v16.0 and R (version 4.3.1; R foundation for Statistical Computing, Vienna, Austria). Medians and interquartile ranges (IQR) of all parameters were calculated for all RV and LV LMS, per medication. To assess normality of continuous parameters, histograms, QQ-plots and Shapiro–Wilk tests were used. In case of normal distribution, a Student’s t-test was used and a Wilcoxon two-sample test otherwise. Differences in biomechanical parameters were tested between RV and LV slices using a linear mixed model, with the ventricle as a fixed effect and patient study number as a random intercept, in order to correct for correlation between slices that were produced from the same patient’s tissue. An analysis comparing non-diseased vs. heart failure RV and LV LMS was performed in the same manner. Non-diseased tissue was defined as tissue derived from hearts of patients without a known history of cardiac disease. Relative changes were calculated by dividing the new value by the baseline value and multiplying this with 100%, and IQRs were calculated from these percentages. A baseline biomechanical contraction profile was constructed for LMS from the RV and the LV before administration of each drug to account for possible changes in baseline profile over cultivation time. For each drug, the percentual increase from baseline to EC_50_ after adding medication was compared between RV and LV LMS. A *p*-value of ≤ 0.05 was considered statistically significant.

## Results

Sixty-three LMS (33 RV and 30 LV) were subjected to a series of titration curves. After applying the exclusion criteria, a total of 369 titration curves (194 RV and 175 LV) could be included for analysis. Baseline characteristics of the patients whose cardiac tissue was used to produce LMS are described in Table 1. Patients had a mean age of 50 ± 19 years and four out of five were male. Two hearts were obtained after explantation for cardiac transplantation, one heart was from a healthy donor whose heart was rejected for transplantation due to technical issues, and two hearts were collected during a lung donation procedure in which the heart was not allocated.

**Table 1:** Patient characteristics

Patient ID	Sex	Age	Reason for tissue donation	Cardiac history	Number of LMS
1	M	59	HTx recipient	Dilated cardiomyopathy	9 (4 RV, 5 LV)
2	M	23	HTx recipient	Acute giant cell myocarditis	16 (8 RV, 8 LV)
3	M	40	LuTx DCD donor	Duchenne’s cardiomyopathy	7 (5 RV, 2 LV)
4	M	52	HTx DCD donor	None	15 (8 RV, 7 LV)
5	F	74	LuTx DBD donor	None	16 (8 RV, 8 LV)

*DBD* donation after brain death, *DCD* donation after circulatory death *HTx,* heart transplant, *LMS* living myocardial slices, *LuTx* lung transplant, *LV* left ventricle, *RV* right ventricle

### Baseline Ventricle-Specific Biomechanical Profile

**Fig. 2:**
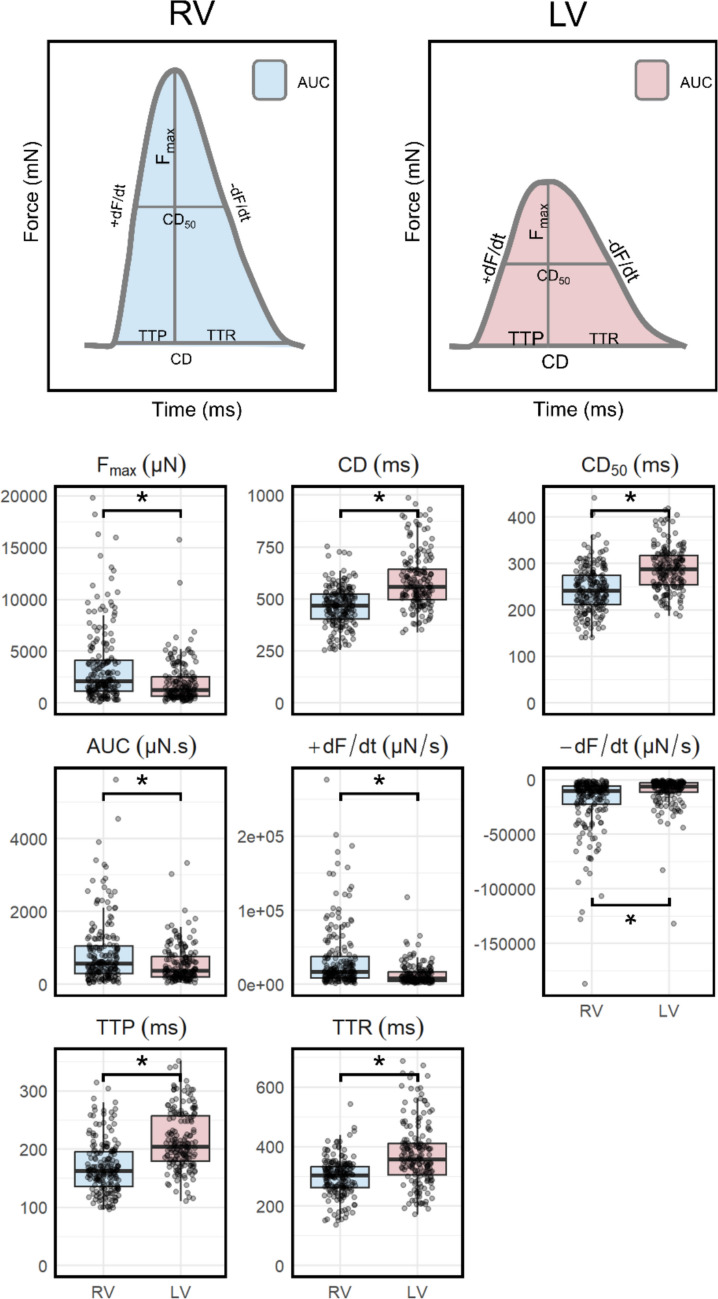
Baseline biomechanical contraction profiles of RV and LV LMS. AUC peak area, CD contraction duration, CD_50_ peak width at 50% of the maximum amplitude, dF/dt slope, F_max_ force amplitude, LV left ventricle, RV right ventricle, TTP time to peak, TTR time to relaxation. * P < 0.001

RV LMS showed a distinct baseline biomechanical contraction profile from the LV LMS (Fig. 2). For RV LMS, the F_max_ was significantly larger, resulting in a steeper + dF/dt and – dF/dt and a larger AUC. The CD, CD_50_, TTP and TTR were significantly shorter for RV than for LV LMS (Fig. 2).

Sensitivity analysis demonstrated no significant differences in baseline contractile parameters between non-diseased and heart failure RV and LV LMS (Supplementary Tables 1–2). Contractile parameters were significantly different at baseline between RV and LV LMS when stratifying LMS derived from non-diseased and heart failure tissue (Supplementary Tables 3–4). These differences were observed in a similar direction in both strata and remained consistent in the combined analysis, as described above.

### Pharmacological Experiments

Dobutamine administration (EC_50_: 3 µM, Supplementary Table 5) resulted in a larger increase in F_max_, dF/dt and TTP in the RV compared to the LV LMS (Table 2). Noradrenaline administration (EC_50_: 0.3 µM) induced a larger decrease in CD_50_ for the LV (RV: 90% (82%—116%) vs. LV: 84% (76%—95%), *p* = 0.03), but no other significant contractile differences (Supplementary Table 6). Enoximone administration (EC_50_: 0.3 µM) also resulted in a slightly but significantly larger increase in CD_50_ between ventricles (RV: 114% (111%—117%) vs. LV: 112% (111%—114%), *p* = 0.049) (Supplementary Table 7). After adrenaline (EC_50_: 0.3 µM) and levosimendan administration (EC_50_: 0.3 µM), no significant differences in biomechanical properties were found between the RV and LV (Supplementary Tables 8–9).

**Table 2:** Relative response to dobutamine administration at EC_50_ (3 µM) compared to baseline in RV vs. LV LMS

Variable	RV	LV	*P*-value
F_max_ (μN)	399% (223–717)	236% (147–429)	0.026*
CD (ms)	79% (70–104)	97% (75–108)	0.14
CD_50_ (ms)	95% (80–111)	97% (90–107)	0.55
AUC (μN.s)	109% (89–123)	107% (95–117)	0.98
+ dF/dt (μN/s)	358% (211–586)	172% (132–229)	0.017*
-dF/dt (μN/s)	458% (193–909)	243% (127–440)	0.021*
TTP (ms)	513% (231–1144)	239% (141–575)	0.016*
TTR (ms)	105% (88–116)	101% (90–130)	0.71

Data are presented as median (IQR). *P ≤ 0.05

*AUC* peak area, *CD* contraction duration, *CD*_*50*_ peak width at 50% of the maximum amplitude, *dF/dt* slope, *F*_*max*_ force amplitude, *LV* left ventricle, *RV* right ventricle, *TTP* time to peak, *TTR* time to relaxation

Dobutamine titration curves could be included from 33 RV LMS and 30 LV LMS

### Force-Frequency Relationship

**Fig. 3:**
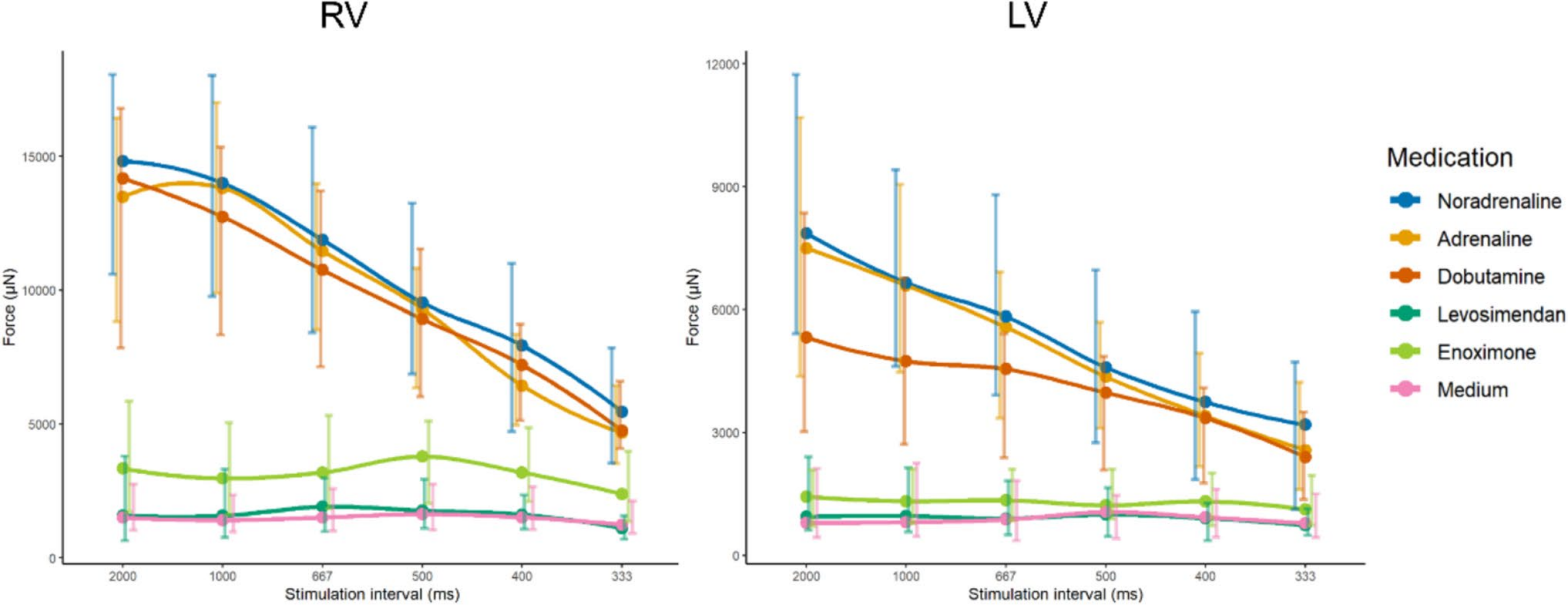
Force-frequency relationship in RV and LV LMS for all medications and medium as control. Data are presented as median (IQR)

A similar pattern was observed for the FFR for noradrenaline and adrenaline in both RV and LV LMS (Fig. 3, Supplementary Table 10). Administration of these drugs resulted in a relatively high F_max_ at the longest stimulation interval of 2000 ms, which steadily decreased with decreasing stimulation intervals, for both ventricles. At the shortest intervals, contractile force of both RV and LV LMS was about a third of the contractile force at 2000 ms. Dobutamine induced a lower F_max_ at 2000 ms in the LV compared to RV LMS, and the decrease in FFR was less steep. The stimulation interval did not affect the force amplitude of LMS receiving enoximone or levosimendan or in the control group (medium). These effects were similar in RV and LV LMS (Fig. 3, Supplementary Table 10).

### Functional Refractory Period

At baseline, the FRP did not differ between RV and LV LMS and the median was 240 (200–300) ms for both. After administration of noradrenaline, the FRP was significantly shorter in RV (220 (200–240) ms) than LV LMS (240 (220–280) ms, *p* = 0.01) (Table 3). Adrenaline showed a similar trend, however this was non-significant (*p* = 0.09). In addition, the median FRP increased more in LV (280 (235–285) ms) than in RV LMS (250 (220–260) ms) after dobutamine administration (*p *= 0.007). No difference in FRP between RV and LV LMS was observed after administration of levosimendan, enoximone, or medium.

**Table 3:** The absolute FRP (FRP_10_) after drug administration and relative change to baseline (ΔFRP) for every medication for both ventricles in ms. The difference in FRP_10_ was tested between ventricles

Medication	RV FRP_10_ (ms)	RV ΔFRP (ms)	*n*	LV FRP_10_ (ms)	LV ΔFRP (ms)	*n*	*P*-value
Adrenaline	220 (200–240)	−40 (−80 to −15)	33	240 (220–260)	−20 (−40 to 0)	27	0.09
Noradrenaline	220 (200–240)	0 (−80 to 30)	32	240 (220–280)	0 (−60 to 20)	29	0.01*
Dobutamine	250 (220–260)	0 (−35 to 20)	32	280 (235–285)	10 (−20 to 40)	28	0.007*
Levosimendan	220 (200–245)	−20 (−45 to 20)	32	200 (180–240)	−40(−50 to −20)	29	0.11
Enoximone	280 (240–340)	20 (0–20)	33	280 (240–340)	0 (0–20)	29	0.93
Medium	260 (220–280)	0 (−20 to 10)	31	250 (205–315)	0 (−20 to 0)	30	0.88

Data are presented as median (IQR)

ΔFRP difference between FRP at 10 µM and baseline, FRP_10 _ FRP determined at a medication concentration of 10 µM, *LMS* living myocardial slices, *LV* left ventricle, *n* number of LMS included for this analysis, *RV* right ventricle

### Irregular Contractions

Irregular contractions occurred significantly more often in the RV (58%) than LV (26%) LMS after adrenaline administration (*p* = 0.03) (Table 4). Noradrenaline showed a similar trend (RV: 65% vs. LV: 39%, *p* = 0.09), but this difference did not reach statistical significance. Irregular contractions were also observed after dobutamine administration, but no significant difference was observed between the two ventricles (RV: 25% vs. LV: 19%, *p*= 0.77). No irregular contractions were observed after administration of levosimendan or enoximone.

**Fig. 4:**
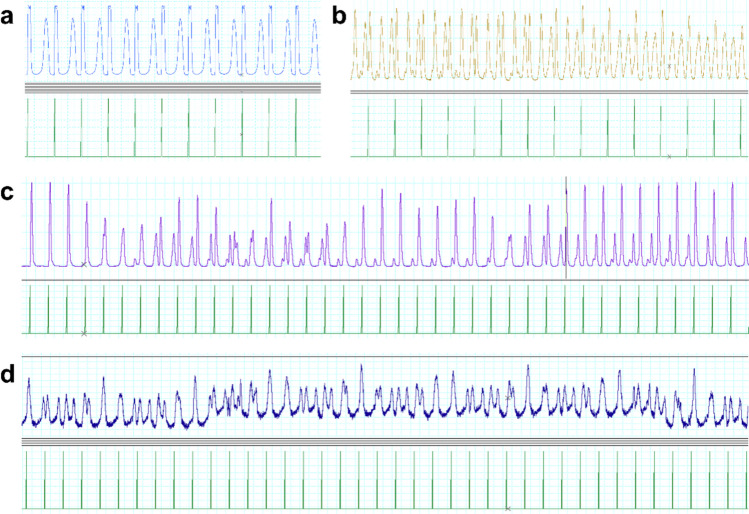
Examples of spontaneous irregular contractions in LMS. The contraction patterns can be described as follows: **a**. Bigeminous contraction pattern; **b**. Irregular contraction pattern transitioning into a trigeminous contraction pattern, consisting of a paced contraction followed by two spontaneous beats; **c**. Temporal regular contraction pattern followed by an irregular contraction pattern transitioning into a bigeminous pattern; **d**. Complete irregular contraction pattern. *The pacing stimulus is illustrated by the green spike*

Examples of irregular contractile patterns can be found in Fig. 4. Regarding the nature of spontaneous contractions, both regular irregularity (e.g., bigeminy) and ‘irregular’ irregularities were observed. A more detailed description of these contraction abnormalities lies outside the scope of this study.

**Table 4:** The occurrence of irregular contractions independent of electrical stimulation at 10 µM

Medication	RV	LV	*P*-value
Adrenaline	19 (58%)	7 (26%)	0.03*
Noradrenaline	20 (65%)	10 (39%)	0.09
Dobutamine	8 (25%)	5 (19%)	0.78
Levosimendan	0 (0%)	0 (0%)	NA
Enoximone	0 (0%)	0 (0%)	NA

*LV *left ventricle*, RV *right ventricle

*χ*^2^-testing could not be performed if irregular contractions were absent

## Discussion

Many differences exist between the RV and LV, and biomechanical analyses specific to the RV are necessary to improve our understanding of RV functionality and enhance treatment of RV dysfunction [1, 15]. Therefore, this study sought to determine the unique biomechanical characteristics of RV and LV human LMS and to compare the response to five commonly used inotropic and vasoactive drugs between both ventricles.

### Key Findings

This study showed that at baseline, RV LMS had a significantly different biomechanical contraction profile, characterised by a larger F_max_ and AUC, steeper + dF/dt and –dF/dt, and shorter contraction durations compared to LV LMS. These differences were observed in both non-diseased and heart failure tissues. For dobutamine, the RV displayed a stronger contractile response than the LV, but for the other drugs, no relevant significant contractile differences between ventricles were observed. After administration of adrenaline, noradrenaline, and dobutamine, spontaneous, irregular contractions were observed in both RV and LV.

### Effect of Heart Failure on LMS Contractility

There were no significant differences between non-diseased and heart failure RV and LV LMS. Although one might expect non-diseased tissue to perform better (e.g., have a higher F_max_) than heart failure tissue, there appeared to be a trend towards the heart failure LMS having a higher F_max_ compared to non-diseased LMS. However, this was not significant, and the comparison could only be made between two non-diseased hearts and three heart failure hearts. One explanation could be that heart failure LMS underwent prior “conditioning” in vivo which confers more with the near physiological state in the BMCC. Previous studies showed that the cardiac energetic metabolism of heart failure patients changes to a more foetal genetic profile with a shift in energetic substrate use from free fatty acids to glucose [1, 16, 17]. Given that the biomimetic culture system uses glucose as energetic substrate, this environment might better suit the heart failure LMS than the non-diseased ones, which could explain why their performance was not worse than that of the non-diseased LMS. Another explanation could be that non-diseased hearts were explanted from circulatory death donors, which might have affected cardiac viability.

### Baseline Differences

At baseline, RV LMS generated a more forceful contraction than LV LMS, with a higher F_max_ and steeper dF/dt resulting in an increased AUC, and within a shorter time period. This contradicts previous studies, which have described the contractile amplitude and unloaded sarcomere shortening of the LV as more pronounced or comparable to the RV [18–22]. According to these studies, the higher contractile amplitudes of the LV could be attributed to increased sarcomere shortening, driven by higher densities of I_Na_ and larger Na^+^ currents, resulting in faster conduction times and altered action potential characteristics [23, 24]. Despite these differences in action potentials between ventricles, excitation–contraction coupling via the Ca^2+^ transient, mediated by activation of L-type Ca^2+^ channel activation, appears unaffected, and no differences in diastolic Ca^2+^ concentrations between LV and RV have been reported at rest [24, 25]. Unfortunately, it was not yet possible to measure real-time Na^+^ and Ca^2+^ transients in the LMS model, so this remains a topic for future research.

These differences in contractile force outcomes between studies may be attributed to differences in the type of experimental model or the source of the studied tissue. Firstly, no previous studies have been performed using human LMS where ventricle-specific micro-architectural features were maintained. Measurements in our study were performed under near-physiological circumstances with preload and stimulation protocols designed to approach the in vivo setting, whereas this was not the case for most other studies [18–24]. Furthermore, most studies used rodent or canine cardiac tissue while numerous studies have already demonstrated that the contractile profile is very different for humans as compared to other animals [26, 27]. Finally, considering the nature of our cardiac specimens, one might argue that the LV tissue had intrinsic failure, as is known to be more prone to (hypoxic) damage than the RV due to its specific morphological features. Nevertheless, our myocardial specimens came from both ventricles of end-stage heart failure patients and non-diseased donors. No significant differences were seen between those groups, implicating that the mechanistic differences are more likely attributed to chamber-specific properties rather than the underlying disease.

RV LMS in this study demonstrate a significantly shorter contraction duration, time to peak and time to relaxation, and steeper + dF/dt and -dF/dt than the LV LMS. These results are supported by earlier reports stating that the RV has a faster contraction velocity during shortening as compared the LV, which was attributed to a higher density of V1 to V3 myosin enzymes [28–30]. The claim of a greater intrinsic velocity of shortening of the RV held true across a wide range of muscle preparations and species [19, 22, 28, 29, 31–33]. Despite this greater intrinsically determined shortening velocity, Pham et al. showed that this did not result in mechano-energetic advantages for the RV as compared to the LV [30].

### Pharmacological Testing: Adrenergic Stimulation

The American Heart Association guideline for treating RV failure recommends using dobutamine or milrinone to augment contractility because of its combined inotropic and vasodilator properties, or an inotropic with a vasopressor effect such as noradrenaline or adrenaline in the setting of hypotension [34]. Our study showed that noradrenaline, adrenaline and dobutamine all increase contractile force in LMS from both ventricles, however, only dobutamine had a significantly different force generation in the RV LMS as compared to the LV LMS. Dobutamine resulted in a larger increase in F_max_, dF/dt and TTP in RV LMS as compared to the LV LMS. It is known that there is a difference in adrenergic responses between RV and LV, and more specifically, in the adaptation of these receptors to different types of pathological loading and heart failure [35–37]. Other studies have already demonstrated ventricular differences in adrenergic receptor expression, possibly explaining part of the differential effects of both ventricles as a result of adrenergic stimulation [38]. Yet, some studies suggest that there are less β-receptors, which are stimulated by dobutamine, in the RV compared to the LV [39, 40]. In that case, the differences in effect may be explained by altered second messenger effectuation. However, since real-time Ca^2+^-handling and second messenger signalling were not directly assessed in this study, the mechanism behind the stronger response to dobutamine in RV LMS remains a topic for further research. From a clinical perspective, the more pronounced response to dobutamine in RV LMS would suggest that dobutamine may be favoured in patients with RV failure. Conversely, in patients with predominant LV failure, a relatively stronger RV response could theoretically lead to pulmonary oedema, which would suggest that dobutamine use warrants caution in these patients [41]. However, the extent to which LMS contractility directly translates to cardiac output remains uncertain, since many other factors are of influence in vivo. Therefore, these potential clinical implications require validation in future studies.

### Pharmacological Testing: Non-Adrenergic Stimulation

Enoximone had no inotropic effect on human LMS in the current study. Levosimendan induced an increase in contractile force that was comparable in both ventricles, but was relatively small when compared to the sympathomimetics. Levosimendan and phosphodiesterase (PDE)3 inhibitors such as enoximone or milrinone are recommended in guidelines for treating RV failure because of their inotropic properties in combination with their ability to reduce cardiac filling pressures [42]. Both drugs have been reported to exert beneficial inotropic effects on the heart through either Ca^2+^ sensitizing mechanisms or PDE inhibition respectively [42, 43], which we could only partially reproduce for levosimendan but not for enoximone in the setting of human LMS. Since the hemodynamic effects can already be observed in human studies after 10–30 min, the positive inotropic effects ascribed to both substances should probably primarily be attributed to extra-cardiac effects [44, 45]. A prior study comparing dobutamine and milrinone found a similar effect of both drugs on RV systolic function and cardiac index in patients with congestive heart failure. However, the authors suggested this was mediated through RV afterload reduction for milrinone, since pulmonary artery end-systolic pressure was significantly reduced for those patients, whereas RV inotropic augmentation contributed more strongly to dobutamine’s effect [46].

### Force-Frequency Relationship

A similar FFR was observed for both ventricles after administration of the different drugs. After administration of noradrenaline, adrenaline and dobutamine, a relatively high F_max_ was observed at a stimulation interval of 2000 ms, which steadily decreased. This was not observed after levosimendan or enoximone administration. These differences in effect on FFR between drugs can possibly be attributed to depletion of ATP within the medium of the biomimetic system for LMS exhibiting stronger contractions, which were those receiving an adrenergic agent.

### Refractory Period and Arrhythmic Properties

A shortening of the FRP promotes early afterdepolarisations, and may therefore serve as a trigger for the occurrence of spontaneous irregular contractions. These irregular contractions occurred twice as often in the RV LMS as in the LV LMS after adrenaline administration, which was a significant difference. A similar, though non-significant, trend was observed for noradrenaline. This finding is in line with the shorter FRP that was observed after noradrenaline and adrenaline administration, with (a trend towards) a larger decrease in FRP in the RV. In contrast to this hypothesis, irregularity also occurred with dobutamine; however, it was observed at the same rate in LMS from both ventricles, even though the FRP was longer for the LV LMS. Clinically, dobutamine is known to have arrhythmogenic properties in both the atria and ventricles, and may induce atrial arrhythmias [47, 48]. Tachyarrhythmias have also been reported as adverse effects of both adrenaline and milrinone [34, 47]. Adrenergic drugs were probably more likely to induce irregular contractions, since they target signalling pathways that alter Ca^2+^ handling inside the cardiomyocyte, thereby increasing excitability. We hypothesize that differences in receptor density between ventricles could explain the higher frequency of irregular, spontaneous contractions in the RV compared to the LV LMS. Furthermore, both regular irregular and ‘irregular’ irregular contraction patterns were observed. However, further classification and the underlying mechanisms are outside the scope of this study.

### Limitations

Firstly, tissue was derived from a heterogeneous group of patients, including those with and without history of cardiac disease. Certain pathology may have influenced our results. However, this allowed us to look into non-diseased and heart failure tissue separately. Secondly, the short time period between titrations may have led to some interactions between drugs, residual drug effects or short-term desensitization, which may have led to an under- or overestimation of the effects, despite the complete medium washout. Moreover, all medications were added in equal concentrations, however clinical target plasma concentrations may differ between drugs. Additionally, the LMS model does not allow to study extracardiac effects of drugs (especially vasopressors). Moreover, whereas the LMS model has a higher complexity and in-vivo mimicry compared to traditional in-vitro models [8], it still lacks the effects of systemic factors such as afterload, neural influence, and hormonal effects. Accordingly, the results of this study reflect direct myocardial responses and may not fully translate into in-vivo hemodynamic effects. Also, no correction for multiple testing was applied in this hypothesis-generating study to limit the risk of a type II error. Nevertheless, this could have led to an overestimation of the reported findings. Furthermore, this study did not investigate the mechanisms underlying the observed differences between ventricles and possible explanations that were mentioned in this study were not directly supported by experimental data, but should be investigated in future studies. Lastly, the BMCC used lacks afterload as present in an in vivo heart and a glucose-based medium was used, which decreases physiological resemblance of the model.

### Conclusions and Future Perspectives

Our study showed a stronger contraction with faster contraction velocity of the RV as compared to the LV in a human living myocardial slices model with a near-physiological state and preload. In addition, it was shown that the RV and LV LMS respond differently to dobutamine, with a stronger response in the RV, being relevant in cardiac decompensated settings. Moreover, spontaneous, irregular contractions were observed after administration of noradrenaline, adrenaline and dobutamine, which occurred significantly more often in the RV after noradrenaline administration. This study highlights the importance of selecting a specific cardiac research model with respect to disease phenotype and ventricular specificities. These findings contribute to the understanding of the RV physiology as a separate entity. The observed differences in biomechanical contraction profile between the ventricles advocate for a different approach in treating RV or LV dysfunction.

## Supplementary Information

Below is the link to the electronic supplementary material.

ESM 1 (DOCX 39.8 KB)

## Data Availability

The data that support the findings of this study are available upon reasonable request to the authors.

## Abbreviations

AUC: Peak area
BMCC: Biomimetic cultivation chamber
CD: Contraction duration
CD_50_: Contraction duration at 50% of the maximum amplitude
EC_50_: The half maximal effective concentration for each medication
FFR: Force frequency relationship
Fmax: Maximum force amplitude
FRP: Functional refractory period
ICU: Intensive care unit
IQR: Interquartile range
LMS: Living myocardial slices
LV: Left ventricle
PDE: Phosphodiesterase
RV: Right ventricle
TTP: Time to peak
TTR: Time to relaxation
+ dF/dt: Steepest positive slope
− dF/dt: Steepest negative slope

## Declarations

### Animal Studies

No animal studies were carried out by the authors for this article.

## References

[1] Taverne YJHJ, Sadeghi A, Bartelds B, Bogers AJJC, Merkus D. Right ventricular phenotype, function, and failure: a journey from evolution to clinics. Heart Fail Rev. 2021;26(6):1447–66. 10.1007/s10741-020-09982-4. 32556672 10.1007/s10741-020-09982-4 PMC8510935

[2] Friedberg MK, Redington AN. Right versus left ventricular failure. Circulation. 2014;129(9):1033–44. 10.1161/CIRCULATIONAHA.113.001375. 24589696 10.1161/CIRCULATIONAHA.113.001375

[3] Ryan JJ, Archer SL. The right ventricle in pulmonary arterial hypertension. Circ Res. 2014;115(1):176–88. 10.1161/circresaha.113.301129. 24951766 10.1161/CIRCRESAHA.113.301129 PMC4112290

[4] Zaffran S, Kelly RG, Meilhac SM, Buckingham ME, Brown NA. Right ventricular myocardium derives from the anterior heart field. Circ Res. 2004;95(3):261–8. 10.1161/01.RES.0000136815.73623.BE. 15217909 10.1161/01.RES.0000136815.73623.BE

[5] Thomas T, Yamagishi H, Overbeek PA, Olson EN, Srivastava D. The bHLH factors, dHAND and eHAND, specify pulmonary and systemic cardiac ventricles independent of left–right sidedness. Dev Biol. 1998;196(2):228–36. 10.1006/dbio.1998.8849. 9576835 10.1006/dbio.1998.8849

[6] Pettersen E, Helle-Valle T, Edvardsen T, Lindberg H, Smith H-J, Smevik B, et al. Contraction pattern of the systemic right ventricle. J Am Coll Cardiol. 2007;49(25):2450–6. 10.1016/j.jacc.2007.02.062. 17599609 10.1016/j.jacc.2007.02.062

[7] Sanz J, Sánchez-Quintana D, Bossone E, Bogaard Harm J, Naeije R. Anatomy, Function, and Dysfunction of the Right Ventricle. J Am Coll Cardiol. 2019;73(12):1463–82. 10.1016/j.jacc.2018.12.076. 30922478 10.1016/j.jacc.2018.12.076

[8] van Doorn ECH, Amesz JH, Sadeghi AH, de Groot NMS, Manintveld OC, Taverne YJHJ. Preclinical models of cardiac disease: a comprehensive overview for clinical scientists. Cardiovasc Eng Technol. 2024. 10.1007/s13239-023-00707-w. 38228811 10.1007/s13239-023-00707-w PMC11116217

[9] Pitoulis FG, Watson SA, Perbellini F, Terracciano CM. Myocardial slices come to age: an intermediate complexity in vitro cardiac model for translational research. Cardiovasc Res. 2020;116(7):1275–87. 10.1093/cvr/cvz341. 31868875 10.1093/cvr/cvz341 PMC7243278

[10] Zhang Y, Li T-S, Lee S-T, Wawrowsky KA, Cheng K, Galang G, et al. Dedifferentiation and Proliferation of Mammalian Cardiomyocytes. PLoS One. 2010;5(9):e12559. 10.1371/journal.pone.0012559. 20838637 10.1371/journal.pone.0012559 PMC2933247

[11] Watson SA, Terracciano CM, Perbellini F. Myocardial Slices: an Intermediate Complexity Platform for Translational Cardiovascular Research. Cardiovasc Drugs Ther. 2019;33(2):239–44. 10.1007/s10557-019-06853-5. 30671746 10.1007/s10557-019-06853-5 PMC6509068

[12] Fischer C, Milting H, Fein E, Reiser E, Lu K, Seidel T, et al. Long-term functional and structural preservation of precision-cut human myocardium under continuous electromechanical stimulation in vitro. Nat Commun. 2019;10(1):117. 10.1038/s41467-018-08003-1. 30631059 10.1038/s41467-018-08003-1 PMC6328583

[13] Watson SA, Scigliano M, Bardi I, Ascione R, Terracciano CM, Perbellini F. Preparation of viable adult ventricular myocardial slices from large and small mammals. Nat Protoc. 2017;12(12):2623–39. 10.1038/nprot.2017.139. 29189769 10.1038/nprot.2017.139

[14] Amesz JH, Langmuur SJJ, Epskamp N, Bogers AJJC, de Groot NMS, Manintveld OC, et al. Acute Biomechanical Effects of Empagliflozin on Living Isolated Human Heart Failure Myocardium. Cardiovasc Drugs Ther. 2023. 10.1007/s10557-023-07434-3. 36780068 10.1007/s10557-023-07434-3 PMC11266265

[15] Gorter TM, van Veldhuisen DJ, Bauersachs J, Borlaug BA, Celutkiene J, Coats AJS, et al. Right heart dysfunction and failure in heart failure with preserved ejection fraction: mechanisms and management. Position statement on behalf of the Heart Failure Association of the European Society of Cardiology. Eur J Heart Fail. 2018;20(1):16–37. 10.1002/ejhf.1029. 29044932 10.1002/ejhf.1029

[16] Koop AC, Bossers GPL, Ploegstra MJ, Hagdorn QAJ, Berger RMF, Sillje HHW, et al. Metabolic Remodeling in the Pressure-Loaded Right Ventricle: Shifts in Glucose and Fatty Acid Metabolism-A Systematic Review and Meta-Analysis. J Am Heart Assoc. 2019;8(21):e012086. 10.1161/JAHA.119.012086. 31657265 10.1161/JAHA.119.012086 PMC6898858

[17] Lopaschuk GD, Karwi QG, Tian R, Wende AR, Abel ED. Cardiac energy metabolism in heart failure. Circ Res. 2021;128(10):1487–513. 10.1161/CIRCRESAHA.121.318241. 33983836 10.1161/CIRCRESAHA.121.318241 PMC8136750

[18] Bernal-Ramirez J, Díaz-Vesga MC, Talamilla M, Méndez A, Quiroga C, Garza-Cervantes JA, et al. Exploring functional differences between the right and left ventricles to better understand right ventricular dysfunction. Oxid Med Cell Longev. 2021;2021(1):9993060. 10.1155/2021/9993060. 34497685 10.1155/2021/9993060 PMC8421158

[19] Švíglerová J, Kuncová J, Nalos L, Holas J, Tonar Z, Rajdl D, et al. Cardiac remodeling in rats with renal failure shows interventricular differences. Exp Biol Med (Maywood). 2012;237(9):1056–67. 10.1258/ebm.2012.012045. 22929800 10.1258/ebm.2012.012045

[20] Sathish V, Xu A, Karmazyn M, Sims SM, Narayanan N. Mechanistic basis of differences in Ca2+-handling properties of sarcoplasmic reticulum in right and left ventricles of normal rat myocardium. Am J Physiol Heart Circ Physiol. 2006;291(1):H88–96. 10.1152/ajpheart.01372.2005. 16461368 10.1152/ajpheart.01372.2005

[21] Kondo RP, Dederko DA, Teutsch C, Chrast J, Catalucci D, Chien KR, et al. Comparison of contraction and calcium handling between right and left ventricular myocytes from adult mouse heart: a role for repolarization waveform. J Physiol. 2006;571(1):131–46. 10.1113/jphysiol.2005.101428. 16357014 10.1113/jphysiol.2005.101428 PMC1805641

[22] Rouleau JL, Paradis P, Shenasa H, Juneau C. Faster time to peak tension and velocity of shortening in right versus left ventricular trabeculae and papillary muscles of dogs. Circ Res. 1986;59(5):556–61. 10.1161/01.Res.59.5.556. 3802429 10.1161/01.res.59.5.556

[23] Calloe K, Aistrup GL, Di Diego JM, Goodrow RJ, Treat JA, Cordeiro JM. Interventricular differences in sodium current and its potential role in Brugada syndrome. Physiol Rep. 2018;6(14):e13787. 10.14814/phy2.13787. 30009404 10.14814/phy2.13787 PMC6046646

[24] Kim JJ, Němec J, Papp R, Strongin R, Abramson JJ, Salama G. Bradycardia alters Ca2+ dynamics enhancing dispersion of repolarization and arrhythmia risk. Am J Physiol Heart Circ Physiol. 2013;304(6):H848–60. 10.1152/ajpheart.00787.2012. 23316064 10.1152/ajpheart.00787.2012 PMC3602772

[25] Bers DM. Cardiac excitation–contraction coupling. Nature. 2002;415(6868):198–205. 10.1038/415198a. 11805843 10.1038/415198a

[26] Milani-Nejad N, Janssen PML. Small and large animal models in cardiac contraction research: advantages and disadvantages. Pharmacol Ther. 2014;141(3):235–49. 10.1016/j.pharmthera.2013.10.007. 24140081 10.1016/j.pharmthera.2013.10.007 PMC3947198

[27] Janssen PML, Periasamy M. Determinants of frequency-dependent contraction and relaxation of mammalian myocardium. J Mol Cell Cardiol. 2007;43(5):523–31. 10.1016/j.yjmcc.2007.08.012. 17919652 10.1016/j.yjmcc.2007.08.012 PMC2093987

[28] Pagani ED, Julian FJ. Rabbit papillary muscle myosin isozymes and the velocity of muscle shortening. Circ Res. 1984;54(5):586–94. 10.1161/01.res.54.5.586. 6723002 10.1161/01.res.54.5.586

[29] Brooks WW, Bing OH, Blaustein AS, Allen PD. Comparison of contractile state and myosin isozymes of rat right and left ventricular myocardium. J Mol Cell Cardiol. 1987;19(5):433–40. 10.1016/s0022-2828(87)80395-4. (S0022-2828(87)80395-4 [pii]). 3625780 10.1016/s0022-2828(87)80395-4

[30] Pham T, Han JC, Taberner A, Loiselle D. Do right-ventricular trabeculae gain energetic advantage from having a greater velocity of shortening? J Physiol. 2017;595(20):6477–88. 10.1113/JP274837. 28857176 10.1113/JP274837 PMC5638877

[31] Han JC, Taberner AJ, Nielsen PM, Loiselle DS. Interventricular comparison of the energetics of contraction of trabeculae carneae isolated from the rat heart. J Physiol. 2013;591(3):701–17. 10.1113/jphysiol.2012.242719. 23184511 10.1113/jphysiol.2012.242719 PMC3577548

[32] Harding SE, O’Gara P, Jones SM, Brown LA, Vescovo G, Poole-Wilson PA. Species dependence of contraction velocity in single isolated cardiac myocytes. Cardioscience. 1990;1(1):49–53. 2102796

[33] McMahon WS, Mukherjee R, Gillette PC, Crawford FA, Spinale FG. Right and left ventricular geometry and myocyte contractile processes with dilated cardiomyopathy: myocyte growth and beta-adrenergic responsiveness. Cardiovasc Res. 1996;31(2):314–23 (0008-6363(95)00212-X [pii].). 8730409

[34] Konstam MA, Kiernan MS, Bernstein D, Bozkurt B, Jacob M, Kapur NK, et al. Evaluation and management of right-sided heart failure: a scientific statement from the American Heart Association. Circulation. 2018;137(20):e578–622. 10.1161/CIR.0000000000000560. 29650544 10.1161/CIR.0000000000000560

[35] Wang G-Y, McCloskey DT, Turcato S, Swigart PM, Simpson PC, Baker AJ. Contrasting inotropic responses to α1-adrenergic receptor stimulation in left versus right ventricular myocardium. Am J Physiol Heart Circ Physiol. 2006;291(4):H2013–7. 10.1152/ajpheart.00167.2006. 16731650 10.1152/ajpheart.00167.2006

[36] Fan T-H, Liang C-s, Kawashima S, Banerjee SP. Alterations in cardiac β-adrenoceptor responsiveness and adenylate cyclase system by congestive heart failure in dogs. Eur J Pharmacol. 1987;140(2):123–32. 10.1016/0014-2999(87)90798-9. 2822436 10.1016/0014-2999(87)90798-9

[37] Wang GY, Yeh CC, Jensen BC, Mann MJ, Simpson PC, Baker AJ. Heart failure switches the RV alpha1-adrenergic inotropic response from negative to positive. Am J Physiol Heart Circ Physiol. 2010;298(3):H913-20. 10.1152/ajpheart.00259.2009. 20035030 10.1152/ajpheart.00259.2009 PMC2838546

[38] Woulfe KC, Walker LA. Physiology of the right ventricle across the lifespan. Front Physiol. 2021;12:642284. 10.3389/fphys.2021.642284. 33737888 10.3389/fphys.2021.642284 PMC7960651

[39] Guillory AN, Clayton RP, Prasai A, El Ayadi A, Herndon DN, Finnerty CC. Biventricular differences in β-adrenergic receptor signaling following burn injury. PLoS One. 2017;12(12):e0189527. 10.1371/journal.pone.0189527. 29232706 10.1371/journal.pone.0189527 PMC5726759

[40] Golf S, Lovstad R, Hansson V. Beta-adrenoceptor density and relative number of beta-adrenoceptor subtypes in biopsies from human right atrial, left ventricular, and right ventricular myocard. Cardiovasc Res. 1985;19(10):636–41. 10.1093/cvr/19.10.636. 2996771 10.1093/cvr/19.10.636

[41] Donker DW, Sallisalmi M, Broomé M. Right-left ventricular interaction in left-sided heart failure with and without venoarterial extracorporeal membrane oxygenation support-a simulation study. ASAIO J. 2021;67(3):297–305. 33627604 10.1097/MAT.0000000000001242 PMC7908866

[42] McDonagh TA, Metra M, Adamo M, Gardner RS, Baumbach A, Böhm M, et al. 2021 ESC Guidelines for the diagnosis and treatment of acute and chronic heart failure: Developed by the Task Force for the diagnosis and treatment of acute and chronic heart failure of the European Society of Cardiology (ESC) With the special contribution of the Heart Failure Association (HFA) of the ESC. Eur Heart J. 2021;42(36):3599–726. 10.1093/eurheartj/ehab368. 34447992 10.1093/eurheartj/ehab368

[43] Szilagyi S, Pollesello P, Levijoki J, Haikala H, Bak I, Tosaki A, et al. Two inotropes with different mechanisms of action: contractile, PDE-inhibitory and direct myofibrillar effects of levosimendan and enoximone. J Cardiovasc Pharmacol. 2005;46(3):369–76. 00005344–200509000–00019 [pii] 10.1097/01.fjc.0000175454.69116.9. 10.1097/01.fjc.0000175454.69116.9 16116344

[44] Lilleberg J, Nieminen MS, Akkila J, Heikkilä L, Kuitunen A, Lehtonen L. Effects of a new calcium sensitizer, levosimendan, on haemodynamics, coronary blood flow and myocardial substrate utilization early after coronary artery bypass grafting. Eur Heart J. 1998;19(4):660–8. 10.1053/euhj.1997.0806. 9597417 10.1053/euhj.1997.0806

[45] Herrmann HC, Ruddy TD, William G, Strauss HW, Boucher CA, Fifer MA. Inotropic effect of enoximone in patients with severe heart failure: demonstration by left ventricular end-systolic pressure-volume analysis. J Am Coll Cardiol. 1987;9(5):1117–23. 2952702 10.1016/s0735-1097(87)80316-9

[46] Eichhorn EJ, Konstam MA, Weiland DS, Roberts DJ, Martin TT, Stransky NB, et al. Differential effects of milrinone and dobutamine on right ventricular preload, afterload and systolic performance in congestive heart failure secondary to ischemic or idiopathic dilated cardiomyopathy. Am J Cardiol. 1987;60(16):1329–33. 10.1016/0002-9149(87)90616-3. 3687783 10.1016/0002-9149(87)90616-3

[47] Tisdale JE, Chung MK, Campbell KB, Hammadah M, Joglar JA, Leclerc J, et al. Drug-induced arrhythmias: a scientific statement from the American Heart Association. Circulation. 2020;142(15):e214–33. 10.1161/cir.0000000000000905. 32929996 10.1161/CIR.0000000000000905

[48] Gianni C, Sanchez Javier E, Mohanty S, Trivedi C, Della Rocca Domenico G, Al-Ahmad A, et al. High-dose dobutamine for inducibility of atrial arrhythmias during atrial fibrillation ablation. JACC: Clinical Electrophysiology. 2020(13). 10.1016/j.jacep.2020.07.018. 10.1016/j.jacep.2020.07.018 33334450

