# Supplementary material for: Ventricle-Specific Biomechanical Responses to Inotropic and Vasoactive Drugs in Human Myocardial Slices
Source: J Cardiovasc Transl Res. 2026 May 11;19(1):53. doi: 10.1007/s12265-026-10773-9 (PMC13160974; doi:10.1007/s12265-026-10773-9)
Supplement: Supplementary file 1 — (DOCX 39.8 KB) [file 12265_2026_10773_MOESM1_ESM.docx]

**Supplementary material**

| **Variable** | **Healthy RV (n=94)** | **Heart failure RV (n=100)** | **P-value** |
| --- | --- | --- | --- |
| F_max_ (μN) | 1646 (914, 3684) | 2848 (1456, 4782) | 0.57 |
| CD (ms) | 443 (373, 493) | 493 (438, 541) | 0.20 |
| CD_50_ (ms) | 222 (195, 247) | 266 (231, 299) | 0.17 |
| AUC (μN.s) | 402 (208, 796) | 710 (416, 1268) | 0.40 |
| +dF/dt (μN/s) | 13960 (6881, 32090) | 19960 (9385, 40485) | 0.90 |
| -dF/dt (μN/s) | -8757 (-21310, -4485) | -12450 (-24060, -6586) | 0.74 |
| TTP (ms) | 155 (127, 171) | 169 (148, 215) | 0.27 |
| TTR (ms) | 286 (237, 321) | 306 (274, 337) | 0.32 |

**Supplementary table 1.** Baseline biomechanical contraction profile parameters for healthy vs. heart failure RV slices.
*AUC: peak area; CD: contraction duration; CD_50_: peak width at 50% of the maximum amplitude; dF/dt: slope; F_max_: force amplitude; LV: left ventricle; RV: right ventricle; TTP: time to peak; TTR: time to relaxation.*

| **Variable** | **Healthy LV (n=87)** | **Heart failure LV (n=88)** | **P-value** |
| --- | --- | --- | --- |
| F_max_ (μN) | 799 (513, 1454) | 1941 (859, 3777) | 0.15 |
| CD (ms) | 564 (502, 676) | 555 (495, 626) | 0.63 |
| CD_50_ (ms) | 273 (251, 309) | 299 (262, 320) | 0.89 |
| AUC (μN.s) | 265 (158, 400) | 599 (286, 1027) | 0.17 |
| +dF/dt (μN/s) | 5217 (3548, 9868) | 12780 (6185, 21780) | 0.15 |
| -dF/dt (μN/s) | -3770 (-7216, -2676) | -8924 (-19250, -3724) | 0.15 |
| TTP (ms) | 195 (178, 223) | 212 (182, 270) | 0.68 |
| TTR (ms) | 359 (316, 465) | 350 (273, 405) | 0.40 |

**Supplementary table 2.** Baseline biomechanical contraction profile parameters for healthy vs. heart failure LV slices.
*AUC: peak area; CD: contraction duration; CD_50_: peak width at 50% of the maximum amplitude; dF/dt: slope; F_max_: force amplitude; LV: left ventricle; RV: right ventricle; TTP: time to peak; TTR: time to relaxation.*

| **Variable** | **RV (n=87)** | **LV (n=94)** | **P-value** |
| --- | --- | --- | --- |
| F_max_ (μN) | 1646 (914, 3684) | 799 (513, 1454) | **<0.001** |
| CD (ms) | 443 (372, 493) | 564 (502, 676) | **<0.001** |
| CD_50_ (ms) | 222 (195.3, 247) | 273 (251, 309) | **<0.001** |
| AUC (μN.s) | 402 (208.3, 796) | 265 (158, 399) | **0.007** |
| +dF/dt (μN/s) | 13960 (6881, 32090) | 5217 (3548, 9868) | **<0.001** |
| -dF/dt (μN/s) | -8757 (-21310, -4485) | -3770 (-7216, -2676) | **<0.001** |
| TTP (ms) | 155 (127, 171) | 195 (178, 223) | **<0.001** |
| TTR (ms) | 286 (237, 321) | 359 (316, 465) | **<0.001** |

**Supplementary table 3.** Baseline biomechanical contraction profile parameters for healthy RV vs. LV slices.
*AUC: peak area; CD: contraction duration; CD_50_: peak width at 50% of the maximum amplitude; dF/dt: slope; F_max_: force amplitude; LV: left ventricle; RV: right ventricle; TTP: time to peak; TTR: time to relaxation.*

| **Variable** | **RV (n=100)** | **LV (n=88)** | **P-value** |
| --- | --- | --- | --- |
| F_max_ (μN) | 2848 (1456, 4782) | 1941 (859, 3777) | **0.001** |
| CD (ms) | 493 (438, 541) | 555 (495, 626) | **<0.001** |
| CD_50_ (ms) | 266 (231, 299) | 299 (262, 320) | **<0.001** |
| AUC (μN.s) | 710 (416, 1268) | 599 (287, 1027) | **0.005** |
| +dF/dt (μN/s) | 19960 (9385, 40485) | 12780 (6185, 21780) | **<0.001** |
| -dF/dt (μN/s) | -12450 (-24060, -6586) | -8924 (-19250, -3724) | **0.006** |
| TTP (ms) | 169 (148, 215) | 212 (182, 270) | **<0.001** |
| TTR (ms) | 306 (274, 337) | 350 (273, 405) | **0.02** |

**Supplementary table 4.** Baseline biomechanical contraction profile parameters for heart failure RV vs. LV slices.
*AUC: peak area; CD: contraction duration; CD_50_: peak width at 50% of the maximum amplitude; dF/dt: slope; F_max_: force amplitude; LV: left ventricle; RV: right ventricle; TTP: time to peak; TTR: time to relaxation.*

| **RV** | | | | | |
| --- | --- | --- | --- | --- | --- |
|  | **0 μM** | **0.3 μM** | **1.0 μM** | **3.0 μM** | **10 μM** |
| **Adrenaline** | 2752 (1161, 4565) | 9953 (5184, 17780)* | 9879 (4740, 17520) | 13626 (8781, 17520) | 14589 (10860, 18440) |
| **Noradrenaline** | 1760 (1126, 3386) | 8767 (3538, 16993)* | 10411 (3020, 18140) | 15134 (6489, 20150) | 15950 (12420, 20300) |
| **Dobutamine** | 1840 (736, 3950) | 3732 (1970, 5935) | 4193 (2409, 7329) | 9738 (5678, 13153)* | 14909 (8565, 18300) |
| **Levosimendan** | 1634 (949, 2741) | 2818 (1242, 4911)* | 3104 (1435, 5096) | 3169 (1665, 4846) | 3048 (1537, 4737) |
| **Enoximone** | 3359 (1463, 6655) | 3890 (2306, 7379)* | 3653 (2031, 6495) | 3527 (2162, 6855) | 3752 (2440, 6421) |
| **LV** | | | | | |
|  | **0 μM** | **0.3 μM** | **1.0 μM** | **3.0 μM** | **10 μM** |
| **Adrenaline** | 1857 (839, 2431) | 7054 (2089, 11588)* | 7893 (2170, 11923) | 8917 (3405, 12000) | 9124 (5103, 11825) |
| **Noradrenaline** | 1055 (713, 1853) | 6283 (2138, 10046)* | 7693 (2980, 11200) | 9135 (4397, 12955) | 9513 (7292, 13140) |
| **Dobutamine** | 1098 (640, 2460) | 1936 (875, 4444) | 1884 (792, 5308) | 4351 (1468, 7064)* | 5817 (3548, 9497) |
| **Levosimendan** | 851 (516, 2716) | 1914 (827, 4042)* | 2100 (890, 4032) | 1653 (895, 3817) | 1800 (814, 4088) |
| **Enoximone** | 1451 (765, 2509) | 2039 (1093, 3655)* | 1778 (1005, 2942) | 1779 (923, 3794) | 1800 (1025, 3311) |

**Supplementary table 5.** Dose-response values for F_max_ (μN) for all medications in RV and LV LMS.
** Used as EC_50_.
EC_50_: the half maximal effective concentration for each medication; LV: left ventricle; RV: right ventricle.*

| **Variable** | **RV (n=32)** | **LV (n=29)** | **P-value** |
| --- | --- | --- | --- |
| F_max_ (μN) | 326% (153, 933) | 387% (211, 660) | 0.41 |
| CD (ms) | 79% (67, 107) | 77% (68, 88) | 0.43 |
| CD_50_ (ms) | 90% (82, 116) | 84% (76, 95) | **0.03*** |
| AUC (μN.s) | 283% (180, 696) | 303% (223, 542) | 0.67 |
| +dF/dt (μN/s) | 523% (132, 1165) | 551% (201, 918) | 0.92 |
| -dF/dt (μN/s) | 477% (137, 870) | 614% (195, 1081) | 0.46 |
| TTP (ms) | 98% (87, 117) | 89% (81, 101) | 0.31 |
| TTR (ms) | 71% (59, 102) | 69% (58, 88) | 0.49 |

**Supplementary table 6.** Relative response to noradrenaline administration at EC_50_ (0.3 µM) in RV vs. LV LMS.
*AUC: peak area; CD: contraction duration; CD_50_: peak width at 50% of the maximum amplitude; dF/dt: slope; F_max_: force amplitude; LV: left ventricle; RV: right ventricle; TTP: time to peak; TTR: time to relaxation.* *P≤0.05

| **Variable** | **RV (n=33)** | **LV (n=28)** | **P-value** |
| --- | --- | --- | --- |
| F_max_ (μN) | 123% (104, 137) | 126% (121, 140) | 0.07 |
| CD (ms) | 113% (111, 116) | 112% (107, 115) | 0.59 |
| CD_50_ (ms) | 114% (111, 117) | 112% (111, 114) | **0.05*** |
| AUC (μN.s) | 137% (123, 155) | 146% (129, 158) | 0.17 |
| +dF/dt (μN/s) | 104% (92, 114) | 109% (99, 116) | 0.12 |
| -dF/dt (μN/s) | 114% (94, 122) | 118% (113, 126) | **0.02*** |
| TTP (ms) | 117% (113, 121) | 115% (109, 120) | 0.42 |
| TTR (ms) | 111% (108, 114) | 109% (105, 116) | 0.38 |

**Supplementary table 7.** Relative response to enoximone administration at EC_50_ (0.3 µM) in RV vs. LV LMS.
*AUC: peak area; CD: contraction duration; CD_50_: peak width at 50% of the maximum amplitude; dF/dt: slope; F_max_: force amplitude; LV: left ventricle; RV: right ventricle; TTP: time to peak; TTR: time to relaxation.* *P≤0.05

| **Variable** | **RV (n=33)** | **LV (n=29)** | **P-value** |
| --- | --- | --- | --- |
| F_max_ (μN) | 305% (139, 719) | 423% (197, 633) | 0.67 |
| CD (ms) | 84% (64, 116) | 73% (67, 95) | 0.56 |
| CD_50_ (ms) | 96% (78, 118) | 84% (75, 90) | 0.11 |
| AUC (μN.s) | 292% (156, 543) | 353% (201, 464) | 0.92 |
| +dF/dt (μN/s) | 387% (127, 1006) | 593% (236, 906) | 0.95 |
| -dF/dt (μN/s) | 410% (136, 1223) | 602% (230, 1090) | 0.89 |
| TTP (ms) | 104% (83, 120) | 91% (80, 111) | 0.10 |
| TTR (ms) | 73% (56, 113) | 73% (58, 93) | 0.76 |

**Supplementary table 8.** Relative response to adrenaline administration at EC_50_ (0.3 µM) in RV vs. LV LMS.
*AUC: peak area; CD: contraction duration; CD_50_: peak width at 50% of the maximum amplitude; dF/dt: slope; F_max_: force amplitude; LV: left ventricle; RV: right ventricle; TTP: time to peak; TTR: time to relaxation.* *P≤0.05

| **Variable** | **RV (n=32)** | **LV (n=29)** | **P-value** |
| --- | --- | --- | --- |
| F_max_ (μN) | 163% (140, 188) | 158% (139, 172) | 0.21 |
| CD (ms) | 108% (102, 113) | 107% (101, 111) | 0.63 |
| CD_50_ (ms) | 110% (106, 113) | 111% (106, 114) | 0.99 |
| AUC (μN.s) | 185% (160, 210) | 170% (144, 189) | 0.28 |
| +dF/dt (μN/s) | 141% (127, 165) | 137% (124, 148) | 0.10 |
| -dF/dt (μN/s) | 154% (132, 171) | 143% (131, 164) | 0.23 |
| TTP (ms) | 112% (109, 119) | 113% (108, 120) | 0.76 |
| TTR (ms) | 104% (99, 115) | 101% (97, 108) | 0.58 |

**Supplementary table 9.** Relative response to levosimendan administration at EC_50_ (0.3 µM) in RV vs. LV LMS.
*AUC: peak area; CD: contraction duration; CD_50_: peak width at 50% of the maximum amplitude; dF/dt: slope; F_max_: force amplitude; LV: left ventricle; RV: right ventricle; TTP: time to peak; TTR: time to relaxation.* *P≤0.05

| **RV** | | | | | | |
| --- | --- | --- | --- | --- | --- | --- |
|  | **2000 ms*** | **1000 ms** | **667 ms** | **500 ms** | **400 ms** | **333 ms** |
| **Adrenaline** | 100% | 98 (91, 102) % | 83 (77, 94) % | 70 (60, 80) % | 56 (42, 67) % | 40 (30, 52) % |
| **Noradrenaline** | 100% | 100 (91, 103) % | 90 (79, 95) % | 73 (62, 83) % | 58 (44, 66) % | 40 (28, 51) % |
| **Dobutamine** | 100% | 94 (87, 99) % | 84 (74, 92) % | 71 (60, 82) % | 58 (45, 70) % | 43 (33, 55) % |
| **Levosimendan** | 100% | 103 (94, 115) % | 105 (89, 140) % | 103 (74, 152) % | 98 (58, 138) % | 55 (36, 124) % |
| **Enoximone** | 100% | 100 (89, 116) % | 104 (80, 141) % | 111 (66, 152) % | 99 (57, 154) % | 72 (41, 136) % |
| **Medium** | 100% | 99 (92, 107) % | 106 (90, 116) % | 107 (87, 122) % | 99 (75, 122) % | 79 (57, 107) % |
| **LV** | | | | | | |
|  | **2000 ms*** | **1000 ms** | **667 ms** | **500 ms** | **400 ms** | **333 ms** |
| **Adrenaline** | 100% | 86 (81, 90) % | 72 (59, 81) % | 62 (46, 71) % | 51 (40, 59) % | 41 (32, 50) % |
| **Noradrenaline** | 100% | 87 (79, 92) % | 76 (63, 83) % | 67 (50, 75) % | 59 (37, 65) % | 48 (25, 56) % |
| **Dobutamine** | 100% | 84 (80, 90) % | 73 (61, 81) % | 64 (48, 74) % | 56 (35, 66) % | 48 (29, 59) % |
| **Levosimendan** | 100% | 96 (88, 102) % | 90 (81, 110) % | 75 (65, 127) % | 67 (46, 129) % | 55 (27, 125) % |
| **Enoximone** | 100% | 105 (94, 116) % | 110 (87, 139) % | 99 (76, 146) % | 99 (63, 142) % | 112 (53, 136) % |
| **Medium** | 100% | 98 (83, 103) % | 97 (69, 120) % | 98 (68, 140) % | 92 (56, 151) % | 76 (51, 166) % |

**Supplementary table 10.** Force-frequency relationship in RV and LV LMS for all medications and medium as control. F_max_ (%) are relative to the F_max_ at the longest stimulation interval.  *LV: left ventricle; RV: right ventricle.* *Reference
